# Response to BNT162b2 mRNA COVID-19 vaccine among healthcare workers in Italy: a 3-month follow-up

**DOI:** 10.1007/s11739-021-02857-y

**Published:** 2021-10-12

**Authors:** Domenico Ponticelli, Fabiana Madotto, Sara Conti, Ippazio C. Antonazzo, Andrea Vitale, Giovanni Della Ragione, Maria L. Romano, Mario Borrelli, Beniamino Schiavone, Riccardo Polosa, Pietro Ferrara, Lorenzo G. Mantovani

**Affiliations:** 1 Pineta Grande Hospital, Castel Volturno, Caserta, Italy; 2 Value-Based Healthcare Unit, IRCCS MultiMedica, Sesto San Giovanni, Milan, Italy; 3 Center for Public Health Research, University of Milan-Bicocca, Via Cadore 48, 20900 Monza, Italy; 4 Center of Excellence for the Acceleration of HArm Reduction (CoEHAR), University of Catania, Catania, Italy; 5 Department of Clinical and Experimental Medicine, University of Catania, Catania, Italy; 6 Health Direction, MultiMedica Group, Milan, Italy

**Keywords:** BNT162b2 vaccine, COVID-19, Healthcare workers, SARS-CoV-2

## Abstract

This study investigated the response to BNT162b2 mRNA COVID-19 vaccine among healthcare workers (HCWs) in an Italian teaching hospital. 444 participants were surveyed with either multiple RT-PCR assays for detection of SARS-CoV-2 nucleic acid in nasopharyngeal swabs or serology testing for the research of virus-specific immunoglobulins. Adverse events following immunization (AEFI) were reported. Two weeks after the first dose anti-SARS-CoV-2 antibodies exceeded reactivity cut-off in 82.5% the participants. Four HCWs tested positive at nasopharyngeal swab after 3 months. More than three-quarters reported AEFIs. Our findings offer an insight regarding the vaccine response after 3 months from its administration, with a special focus on effectiveness data, as well as the type and number of AEFIs complained by HCW recipients. The presented study may serve as reference for future research which will be necessary to explore the long-term safety of this vaccine, especially in population at high risk for infection, such as HCWs.

## Introduction

Effective and timely immunologic response to vaccines is a crucial strategy for the control of the severe acute respiratory syndrome—coronavirus 2 (SARS-CoV-2) pandemic. The two-dose messenger RNA (mRNA) vaccine BNT162b2 showed an efficacy of 94.8% against novel coronavirus disease 2019 (COVID-19). Its immune response is direct against the SARS-CoV-2 S1 spike protein, and antibody titers are associated with functional virus neutralization [1].

After a positive assessment of safety and efficacy for BNT162b2 mRNA vaccine granted by different national and international regulatory agencies, a global vaccination campaigns targeted healthcare workers (HCWs) as the first vaccinees, both for ensuring their protection and safety at work, and the sustainability of healthcare systems [2, 3]. Growing evidence is emerging about the vaccine response, but data on effectiveness and safety after the second dose deserve careful evaluation [4]. In particular, real-world accurate information regarding adverse events following immunization (AEFI) is important to educate the population and to foster vaccination campaign itself. Therefore, we assessed the response to BNT162b2 mRNA COVID-19 vaccine in a sample of HCWs of an Italian teaching hospital.

## Methods

Here, we present the results of a 3-month longitudinal observational study conducted among the healthcare workers (HCWs) of the Pineta Grande Hospital (Castel Volturno, Caserta, Italy) with the objective of evaluating the response to the two-dose mRNA vaccine BNT162b2 administered between December 2020 and January 2021.

All the internal HCWs were invited to participate in the study before the administration of the vaccine, performed according to the manufacturer’s instruction as a two-dose regimen with an interval of 3 weeks. The participation was voluntary, vaccinees were not offered any incentive, and were informed about their right to withdraw at any time without penalty. The vaccinees were interviewed about their demographics and professional characteristics (sex, age, professional role, hospital unit, history of symptoms compatible with COVID-19, previous laboratory-confirmed SARS-CoV-2 infection). They were also asked to report any local (pain, redness, and swelling injection site) and systemic AEFI (fatigue, headache, muscle and joint pain, chills, fever, swelling of the face, tongue, throat, breathing problems) occurred within 7 days after the prime and booster doses. According to the study protocol, vaccinees were randomly divided in two groups, to undergo, respectively, (A) six nasopharyngeal swabs and real-time polymerase chain reaction (RT-PCR) assays for qualitative detection of SARS-CoV-2 nucleic acid; (B) or quantitative serology testing for the research of virus-specific immunoglobulins (Ig). The first tests were taken before the administration of the vaccine (t_0_) and then according to a predefined timeline (at 15, 30, 45, 60, and 90 days from the first dose). Seroprevalence was assessed ADVIA Centaur® XPT Immunoassay System for the research of SARS-CoV-2 antibodies (Siemens Healthcare GmbH, Munich, Germany) in serum samples, and reactivity was intended as antibody level Index ≥ 1.0, according to manufacturer’s instructions. Study protocol included up to six longitudinal serological tests, but tests were stopped upon the achievement of the maximum Index value of 10.

SARS-CoV-2 infection was traced through RT-PCR on samples obtained from nasopharyngeal swabs, according to manufacturer’s instructions. A passive surveillance follow-up for potential SARS-CoV-2 infections was set among the vaccinees after the end of the study.

A descriptive analysis was carried out to describe cohort characteristics and outcomes. Continuous variables were expressed as mean and standard deviation (SD), or median and interquartile range (IQR), according to their distribution. Categorical variables were described as absolute and relative frequency. Multivariate logistic regression analyses were built to investigate the association between the probability of seroconversion and AEFIs occurrence, and potential predictors, namely sex, age, and previous SARS-CoV-2 infection. Results were reported as adjusted odds ratios (OR_adj_) and 95% confidence intervals (CI). All statistical tests were two tailed and a *p* value ≤ 0.05 was considered statistically significant.

## Results

Overall, 444 HCWs participated in this study: the characteristics of the study participants and their distribution into the two groups is presented in Table 1.

**Table 1:** Study population

Study population	*N* (%)
Total vaccinees	444
Age, mean ± SD	37.9 ± 12.1
Sex (male)	197 (44.4)
Previous SARS-CoV-2 infection	43 (9.7)
In close contact with patients	350 (78.8)
Healthcare workers	
Physician	54 (12.2)
Nurse	197 (44.4)
Other HCWs	29 (6.5)
Students	74 (16.7)
Others	90 (20.3)
Ward	
Medical	86 (19.4)
Surgical	114 (25.7)
Other medical	70 (15.8)
Nurse students	74 (16.7)
Administrative	100 (22.5)

Cohort of subjects who underwent blood test for detecting SARS-CoV-2 antibodies	*N* (%)	Cohort of subjects who underwent SARS-CoV-2 swab	*N* (%)
Cohort	126	Cohort	318
Age, mean ± SD	40.7 ± 11.1	Age, mean ± SD	36.9 ± 12.3
Sex (male)	49 (38.9)	Sex (male)	148 (46.5)
Previous SARS-CoV-2 infection	7 (5.6)	Previous SARS-CoV-2 infection	36 (11.3)
In close contact with patients	82 (65.1)	In close contact with patients	268 (84.3)
Subjects with positive serological test at the enrollment	13 (10.3)	Time (days) between the first vaccine dose and swabs, median [IQR]	
		I swab	15 [13–16]
		II swab	30 [29–32]
Time (days) between 1st the first vaccine dose and blood serological tests, median [IQR]		III swab	46 [44–47]
I blood test	14 [13–16]	IV swab	60 [59–61]
II blood test*	30 [29–31]	V swab	91 [89–93]
III blood test°	44 [43–45]	Positive result to swab	
Cumulative probability of reactivity (≥ 1)		I swab	6 (1.9)*
Time 1	104 (82.5)	II swab	1 (0.3)*
Time 2	126 (100)	III swab	0 (0.0)
Time 3	126 (100)	IV swab	0 (0.0)
Cumulative probability of reactivity (> 10), *n* (%)		V swab	4 (1.3)*
Time 1	31 (26.6)		
Time 2	124 (98.4)		
Time 3	126 (100)		
Antibody levels, median [IQR]			
Time 1	2.9 [1.2–9.6]		
Time 2	> 10 [> 10–> 10]		
Time 3	> 10 [> 10–> 10]		

*SARS-CoV-2* severe acute respiratory syndrome—coronavirus 2; *HCW* healthcare worker; *SD* standard deviation*; IQR* interquartile range

*All in close contact with patients

In the cohort A, six subjects tested positive at nasopharyngeal swabs within 15 days after the first vaccine dose and one about a week from the second dose. At 45- and 60-day follow-ups all vaccinees tested negative, but four positive tests were registered at the third month. Overall, two were asymptomatic and nine mildly symptomatic infections. In the cohort B, at 2 weeks after the first vaccine dose anti-SARS-CoV-2 antibodies exceeded the reactivity cut-off in 82.5% of the participants. At one-month follow-up, almost all (98.4%) the vaccinees had reached the maximum Index value of 10. The likelihood of reaching this level at two-week follow-up was higher in younger HCWs (OR_adj_ 0.95 *per annum*; 95% CI 0.91–0.99; *p* = 0.03) and in those who had a baseline antibody level greater than the reactivity threshold (OR_adj_ 13.18; 95% CI 3.30–52.71; *p* < 0.001). In the whole sample (*n* = 444), one more case was reported to the in-hospital passive surveillance system in the third month after the end of the study.

AEFIs were studied in the whole sample. 75.5% and 80.0% the participants self-reported at least one AEFI after the first and the second dose, respectively. The most frequent AEFIs were local reactions at injection site. Among the participants, no life-threatening AEFIs were reported. No hospitalizations or visits to emergency department were observed in the study period. A complete AEFIs overview is reported in Table 2: they were found to be heterogeneously associated with sex and age. Fatigue, headache, muscle and joint pain, and fever after the first dose were more likely to be reported by subjects with a prior SARS-CoV-2 infection (OR_adj_ 2.19; 95% CI 1.12–4.28; *p* = 0.02). No significant association was found between the occurrence of any AEFIs at second dose and SARS-CoV-2 infection.

**Table 2:** Adverse effects reported by HCW vaccinees (*n* = 444)

Adverse effect		
After the first dose		After the second dose
335 (75.5)*	*At least one*	355 (80.0)*
285 (64.2)*	Pain, redness, and swelling injection site	271 (61.0)*
168 (37.8)*	Fatigue	248 (55.9)*
126 (28.4)**	Headache	215 (48.4)*
119 (26.8)**	Muscle pain	198 (44.6)*
90 (20.3)**	Chills	173 (39.0)**
83 (16.7)**	Joint pain	148 (33.3)
25 (5.63)	Fever	111 (25.0)*
8 (1.8)	Enlarged lymph nodes	26 (5.9)
2 (0.5)	Urticaria	4 (0.9)
7 (1.6)	Swelling of face, tongue, or throat	6 (1.4)
3 (0.7)	Breathing problems	2 (0.5)
11 (2.5)	Others	9 (2.0)

*Association with sex (female) and age (younger age) (*p* < .05)

**Association with sex (female) (*p* < .05)

## Discussion

Real-world data from this study offer an interesting insight regarding the vaccine response after 3 months from the administration with BNT162b2 vaccine. 82.5% the participants showed clinical markers of immunologic response after the first BNT162b2 dose (second week) and 1 week after the second doses, almost all (98.4%) the vaccinees developed anti-SARS-CoV-2 antibodies at the maximum Index value of 10. As expected, anti-S antibody level was boosted by the second dose, which consequentially increased the functional protection against SARS-CoV-2/COVID-19 [1, 5]. More interestingly, we found that vaccine-induced antibody level was significantly high after 2 weeks from the single dose of SARS-CoV-2 mRNA vaccine in HCWs with preexisting immunity. Several data showed that one dose of the BNT162b2 vaccine (as well as other COVID-19 vaccines) maximizes the cellular and humoral immune response to S1 spike protein in subjects with previous SARS-CoV-2 infection and our results corroborate the hypothesis that previous SARS-CoV-2 infection may be considered an analogous to immune priming [6, 7].

COVID-19 cases have been reported in people who received one or both doses of the vaccine [1, 8, 9]. Four SARS-CoV-2 infections were registered at third month, with the development of a mild COVID-19-like symptomatology. Overall, the proportion of testing positive for SARS-CoV-2 after vaccination was 1.3%, in line with a previous survey among HCWs [4, 9] but slightly higher than that registered among BNT162b2-trial participants. In particular, Keehner and coll highlighted important differences between the HCW populations and BNT162b2-trial participants, such as the availability of regular testing—irrespectively of symptoms’ occurrence—and the younger age of the HCWs compared with the trial participants [1, 9]. It should be also remarked the higher risk of SARS-CoV-2 exposure for hospital workers compared with the general population [2, 3]. In addition, it is possible that those individuals with a PCR-positive swab before day 15 may not have already developed an efficacious immune response. In this regard, a study investigating the initial impact of SARS-CoV-2 vaccination on HCWs in Italy found that the reduction in the proportion of cases was only observed starting from around 30 days after the start of the vaccination campaign [4], likely depending on neutralizing antibody titers during the peri-infection period [10]. Interestingly, in the trimester after the end of the study period, only one SARS-CoV-2 case was registered at the surveillance follow-up.

As regards AEFIs occurrence, almost all the referred events were mild symptoms, with a vast majority of common local reactions to vaccines (pain, redness, or swelling injection site). No AEFIs other than the expected were observed, with a few suspected allergic reactions, confirming the safety profile of the BNT162b2 COVID-19 vaccine. Interestingly, the findings from this study showed that sex and age could affect AEFIs, with a higher likelihood in women and younger vaccinees. Evidence on sex-based differences in vaccine response has been previously described. Women showed a stronger immune response to vaccines that might enhance their effectiveness. On the other hand, this more effective innate and adaptive immune response might make women also more prone to experience AEFIs following vaccines administration [11]. Although a possible explanation of this phenomenon has been proposed, more sex-disaggregated data on COVID-19 vaccines are needed in order to deeply understand these sex differences. Similarly, younger age is associated to more a robust immune response, which could explain the higher AEs incidence compared with older recipients [12].

Some first-dose AEFIs—namely, fatigue, headache, muscle and joint pain, and fever—were associated with previous reports of laboratory-confirmed COVID-19, but at the second administration there was no difference in AEFIs occurrence between these recipients and those with no history of COVID-19. Larger studies should investigate duration and severity of AEFIs following mRNA vaccination in association with previous SARS-CoV-2 infection [6–9].

It is important to point out the main limitations of the presented study. The type of immunoassay system used for the research of SARS-CoV-2 antibodies in blood samples did not allow the study of the time trend of antibody levels in vaccine recipients; however, this was not intended as research objective. Again, although the most reported AEFIs are generally associated with vaccine administration, the presence of potential notoriety bias (defined as “a selection bias in which a case has a greater chance of being reported if the subject is exposed to the studied factor known to cause, thought to cause, or likely to cause the event of interest” [13]), with consequent over-reporting of certain AEFIs, cannot be completely excluded.

Despite these limitations, this research adds useful data to the current growing evidence on response to and safety of COVID-19 vaccines. It also offers direct practical implications that could contribute to refine the planning for future boosters to keep people protected. Lastly, this study focusses on AEFI incidence, offering important information that may refine current estimates of AEFI data and could positively contribute to vaccination acceptance.

In conclusion, this research adds important real-world data on the ongoing vaccination campaigns against COVID-19, the most valuable effective and safe strategy to protect the population against the disease. The low number of SARS-CoV-2 infections 3 months after administration of the second dose of vaccine is an important confirmation of the effectiveness of BNT162b2 mRNA COVID-19 vaccine, suggesting that it is maintained over time and among subjects at high risk for the infection. Longer follow-ups are needed to investigate the duration of the protection and time trends of antibody level, as well as to provide evidence-based metrics that may help maximize the current global vaccination campaign.

## Data Availability

Data and supporting materials associated with this study will be provided upon request by contacting the corresponding author.

## References

[1] Polack FP, Thomas SJ, Kitchin N (2020). Safety and efficacy of the BNT162b2 mRNA COVID-19 vaccine. N Eng J Med.

[2] Della Valle P, Fabbri M, Madotto F (2021). Occupational exposure in the Lombardy region (Italy) to SARS-CoV-2 infection: results from the MUSTANG–OCCUPATION–COVID-19 study. Int J Environ Res Public Health.

[3] Ferrara P, Albano L (2020). COVID-19 and healthcare systems: what should we do next?. Public Health.

[4] Mateo-Urdiales A, Del Manso M, Andrianou X (2021). Initial impact of SARS-Cov-2 vaccination on healthcare workers in Italy—Update on the 28th of March 2021. Vaccine.

[5] Krammer F, Srivastava K, Alshammary H (2021). Antibody responses in seropositive persons after a single dose of SARS-CoV-2 mRNA vaccine. N Eng J Med.

[6] Manisty C, Otter AD, Treibel TA (2021). Antibody response to first BNT162b2 dose in previously SARS-CoV-2-infected individuals. Lancet.

[7] Buonfrate D, Piubelli C, Gobbi F (2021). Antibody response induced by the BNT162b2 mRNA COVID-19 vaccine in a cohort of health-care workers, with or without prior SARS-CoV-2 infection: a prospective study. Clin Microbiol Infect.

[8] Dagan N, Barda N, Kepten E (2021). BNT162b2 mRNA Covid-19 vaccine in a nationwide mass vaccination setting. N Engl J Med.

[9] Keehner J, Horton LE, Pfeffer MA, et al. SARS-CoV-2 Infection after Vaccination in Health Care Workers in California N Engl J Med. 2021 May 6;384(18):1774–1775. 10.1056/NEJMc2101927 PMC8008750 33755376

[10] Bergwerk M, Gonen T, Lustig Y (2021). COVID-19 breakthrough infections in vaccinated health care workers. N Engl J Med.

[11] McCartney PR (2020). Sex-based vaccine response in the context of COVID-19. J Obstet Gynecol Neonatal Nurs.

[12] Jeon M, Kim J, Oh CE, Lee J (2021). Adverse events following immunization associated with coronavirus disease 2019 vaccination reported in the mobile vaccine adverse events reporting system. J Korean Med Sci.

[13] Bégaud B, Dangoumau J (2000). Pharmacoepidemiology: definitions, problems, methodology. Therapie.

